# Non-PGM Electrocatalysts for PEM Fuel Cells: A DFT Study on the Effects of Fluorination of FeN_x_-Doped and N-Doped Carbon Catalysts

**DOI:** 10.3390/molecules26237370

**Published:** 2021-12-04

**Authors:** Mohamed Cherif, Jean-Pol Dodelet, Gaixia Zhang, Vassili P. Glibin, Shuhui Sun, François Vidal

**Affiliations:** Centre Énergie, Matériaux, Télécommunications, Institut National de la Recherche Scientifique, 1650 Bd. Lionel-Boulet, Varennes, QC J3X 1S2, Canada; mohamed.cherif@inrs.ca (M.C.); jean-pol.dodelet@inrs.ca (J.-P.D.); gaixia.zhang@inrs.ca (G.Z.); vassili.glibin@gmail.com (V.P.G.); shuhui.sun@inrs.ca (S.S.)

**Keywords:** oxygen reduction reaction, proton exchange membrane fuel cell, fluorination, density functional theory, non-noble metal catalyst, N-doped carbon catalyst

## Abstract

Fluorination is considered as a means of reducing the degradation of Fe/N/C, a highly active FeN_x_-doped disorganized carbon catalyst for the oxygen reduction reaction (ORR) in PEM fuel cells. Our recent experiments have, however, revealed that fluorination poisons the FeN_x_ moiety of the Fe/N/C catalytic site, considerably reducing the activity of the resulting catalyst to that of carbon only doped with nitrogen. Using the density functional theory (DFT), we clarify in this work the mechanisms by which fluorine interacts with the catalyst. We studied 10 possible FeN_x_ site configurations as well as 2 metal-free sites in the absence or presence of fluorine molecules and atoms. When the FeN_x_ moiety is located on a single graphene layer accessible on both sides, we found that fluorine binds strongly to Fe but that two F atoms, one on each side of the FeN_x_ plane, are necessary to completely inhibit the catalytic activity of the FeN_x_ sites. When considering the more realistic model of a stack of graphene layers, only one F atom is needed to poison the FeN_x_ moiety on the top layer since ORR hardly takes place between carbon layers. We also found that metal-free catalytic N-sites are immune to poisoning by fluorination, in accordance with our experiments. Finally, we explain how most of the catalytic activity can be recovered by heating to 900 °C after fluorination. This research helps to clarify the role of metallic sites compared to non-metallic ones upon the fluorination of FeN_x_-doped disorganized carbon catalysts.

## 1. Introduction

While promising non-noble metal catalysts for the oxygen reduction reaction (ORR) in proton-exchange membrane (PEM) hydrogen fuel cells have been synthesized over the years [1,2,3,4,5], their stability in fuel cells remains the main obstacle to their widespread use [6,7]. One of the most promising non-noble metal catalysts synthesized to date is FeN_x_-doped disorganized carbon [8,9,10,11]. There are several experimental and theoretical pieces of evidence that the Fe atom is the site where the ORR takes place [11,12,13,14,15,16,17,18,19]. It has been observed that this type of catalyst suffers from a decrease of almost half of its activity in a few hours of operation in fuel cells, followed by a much slower decrease thereafter. The current delivered by the fuel cell versus time can be fitted by a double exponential decay [20]. Few hypotheses have been put forward to explain the first rapid decay of catalytic activity. These include demetallation of the metal catalytic sites [20,21,22,23] and chemical reactions with H_2_O_2_ [24,25,26,27]. The slower decay has not attracted as much interest as the fast one to date. Recent simulation work suggests that planar M_3_(C_6_O_6_)_2_ [28] and M_3_(C_6_S_3_O_3_)_2_ [29] structures, where M is a transition metal, may also be promising candidates but these have not yet passed the test of experiment.

There are indications that the fluorination of materials in acidic media improves their oxidative stability. Examples of such systems include Nafion ionomer, Pt/C, and platinum group metal-free catalysts for PEM fuel cells [30,31,32]. Recently, we also fluorinated a highly active FeN_x_-doped carbon catalyst in the hopes that fluorine would increase its stability in PEM fuel cells [33]. However, even after a short (2 min) exposure to a room-temperature F_2_:N_2_ (1:1; *vol.*) gas stream, fluorination considerably inhibited the catalyst performance in H_2_/O_2_ PEM fuel cells. Even if these experiments did not yield the expected results, they enabled several important observations to be made regarding the properties of the catalytic material under study:

(1) The catalytic activity of the fluorinated Fe/N/C catalyst became similar to that of Fe-free nitrogen-doped carbon catalysts;
(2) The XPS F1s spectra revealed that most Fe sites were associated with a single F atom and fewer were associated with two F atoms;
(3) A total of 70% of the initial activity could be recovered after a heat treatment of the F-poisoned catalyst at 900 °C in Ar.

The observations made in the context of these fluorination experiments have in fact provided a unique opportunity to improve our understanding of the nature of our FeN_x_-doped carbon catalysts and of the decay mechanisms of their catalytic activity in PEM fuel cells. In order to support and deepen the conclusions of our experimental study, in this paper we report density functional theory (DFT) calculations, based on the current understanding of the atomic structure of the catalytic sites and processes, and study the catalytic properties of these sites in the absence/presence of adsorbed fluorine.

Several theoretical studies have already focused on MN_x_-doped carbon catalysts, most often Fe [34,35,36,37,38], Co [35], Mn [35,39], and Ni [35]. Per the indications of several experimental studies [34,39,40,41], they conclude that the catalytic site is, specifically, the M atom within a functional group MN_x_ embedded in a planar carbon layer. It is generally thought that the ORR catalyzed on these sites follows the four-electron exchange process [42,43,44,45]

(1) $$\begin{array}{c} {}*+O_{2}+4\left(H^{+}+e^{-}\right)\\ I \end{array}\begin{array}{c} \;\;\;\;\to \;\;\;\;*O_{2}+4\left(H^{+}+e^{-}\right)\;\;\to \\ \;\;\;\;\;\;\;\mathrm{II} \end{array}\;\begin{array}{c} {}*\;\mathrm{OOH}+3\left(H^{+}+e^{-}\right)\\ \mathrm{III} \end{array}\begin{array}{c} \;\;\;\;\to \;\;\;\;*O+H_{2}O+2\left(H^{+}+e^{-}\right)\;\;\to \\ \mathrm{IV} \end{array}\;\begin{array}{c} \;\;\;\;\;\;\;\;\;\;\;\;*\mathrm{OH}+H_{2}O+\left(H^{+}+e^{-}\right)\\ \;\;\;\;\;\;\;\;\;\;\;\;V \end{array}\begin{array}{c} \;\;\;\;\to \;\;\;\;*+2H_{2}O\\ \;\;\;\;\;\;\;\;\;\;\mathrm{VI} \end{array}$$

where * denotes the adsorption site and the labels I to VI refer to the six reaction steps.

For several MN_x_-doped carbon structures, it has been found that, at low enough potentials, the free energy of each step of the reaction sequence (1) decreases uniformly from the first to the last step, indicating that the reaction sequence (1) is thermodynamically viable at these potentials. Other possible pathways, such as those involving spontaneous O_2_ dissociation or H_2_O_2_ formation, are less likely due to the increase in free energy at some stage of the process [24]. Several DFT studies have also been carried out for catalysts without metal [45,46,47,48,49,50,51]. These generally consider N-doped carbon structures and assume that the reaction sequence (1) still takes place at low enough potentials. These catalysts appear to be thermodynamically viable for some carbon sites near a nitrogen atom. However, O_2_ adsorbs weakly or not at all on the catalytic sites (step II in the sequence (1)). This characteristic likely explains, at least in part, the much lower activity of these sites compared to the higher activity obtained with metal sites.

In a recent work, we theoretically studied the fluorination of two single-layer porous FeN_4_-doped carbon structures, one with pyrrolic nitrogen atoms and the other with pyridinic nitrogen atoms at the FeN_4_ sites, and we assumed that the catalytic reaction took place through the sequence (1) [52]. Subsequent work has investigated ORR for various adsorbates bound to several transition metals on an MN_4_ site [53,54]. However, actual catalysts most likely contain many embodiments of MN_x_ moieties as well as M-free N atoms in carbon layers, and involve more than one carbon layer. In order to provide a more complete picture of the fluorination process and of its influence on ORR, we performed DFT calculations for nine additional atomic structures of FeN_x_-doped carbon sites with x between 1 and 4 as well as for two N-doped metal-free carbon structures. We also examined the possibility of whether the ORR can be catalyzed on an Fe site between two parallel carbon layers in the presence of a F atom bound on Fe on the opposite side.

**Figure 1:**
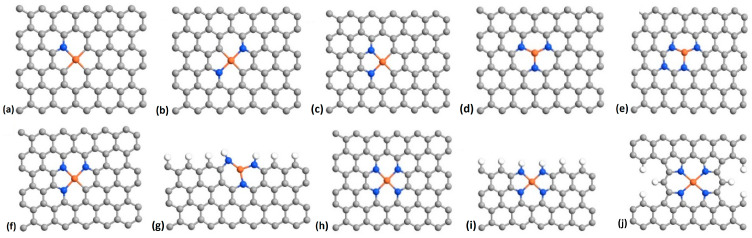
DFT optimized configurations of FeN_x_-doped carbon catalysts investigated in this work. Panels (**a**–**j**) refer to the 10 configurations considered in this work. Color code: grey is carbon, blue is nitrogen, orange is iron.

It is generally believed that the catalytic sites for the ORR of the FeN_x_-doped carbon materials consist of planar FeN_x_ moieties located in a carbon structure which is generally approximated by a single carbon layer. Figure 1 shows some of the possible embodiments of such structures. Of course, the set of structures shown in Figure 1 is not exhaustive. Many variants of each structure are possible, such as, for example, pores in the carbon layer (as in Figure 1j), the FeN_x_ moiety being near the edge of the carbon layer (as in Figure 1g,i), and N atoms being randomly distributed in the carbon layer (as in Figure 1e). Also, the N atoms surrounding the Fe ion may be either of a pyridinic type (Figure 1a–i) or of a pyrrolic type (Figure 1j). We will consider the 10 structures shown in Figure 1, expecting that they will be representative of the main effects of fluorination on the properties of the catalysts. For the purpose of the following discussion, special attention will be paid to the structure in Figure 1j. For this structure, the F–N bond length is 2.00 Å and the N–Fe–N angles are 197.19° and 82.81°.

It can be found in the literature that the free energy at zero potential of the steps of the reaction sequence (1) for the pristine structures of Figure 1c [55], 1h [56], and 1j [52] is uniformly descending at each reaction step (see Section 2.1). The structure of Figure 1b has also been investigated and was found to be not thermodynamically viable [55] (because the free energy presents a minimum at step V of the reaction sequence (1)—see Section 2.1). The other sites shown in Figure 1 have, to the best of our knowledge, never been investigated so far.

**Figure 2:**
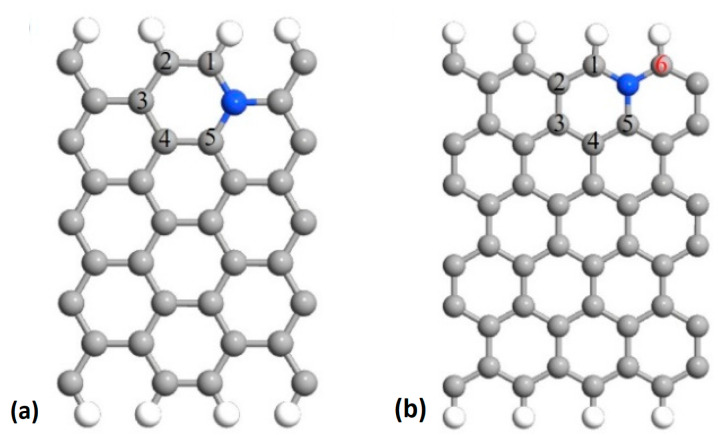
DFT optimized configurations of metal-free catalysts investigated in this work. (**a**) Armchair configuration of N-doped carbon and (**b**) zigzag configuration of N-doped carbon. The meaning of the numbers on the carbon atoms is discussed in Section 2.3.

Figure 2 shows the two metal-free nitrogen-doped carbon catalytic sites considered in this work. As reported above, the activity of the fluorinated Fe/N/C catalysts became similar to that of Fe-free nitrogen-doped carbon catalysts. It is assumed that the reaction sequence is given by (1) where * now denotes an active carbon site. Other nitrogen-doped carbon structures have been investigated and shown to be potential catalysts for ORR [46,47,48,49]. The structures shown in Figure 2 were selected for this work because they turn out to have the lowest formation energies [45] and are, therefore, most likely to be found in actual catalysts. Only two of these structures are considered in this work because we show that their catalytic properties are immune to fluorination and this result is sufficient for our purpose.

## 2. Results

### 2.1. Fluorination of the FeN_x_ Sites—Single Carbon Layer

We first performed DFT optimizations of all the non-fluorinated carbon-based catalysts considered in this work. The resulting structures are shown in Figure 1 and Figure 2. We then introduced F atoms and F_2_ molecules at different locations on these atomic structures and performed new DFT optimizations.

**Figure 3:**
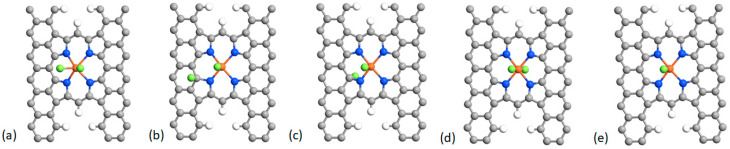
Adsorption of F_2_ and F on the Fe site for the basic structure of Figure 1j. (**a**) Adsorption of F_2_; (**b**,**c**) adsorption of one F on Fe and one F on a nearby carbon site; (**d**) adsorption of two F on the Fe site on both sides of the carbon layer; and (**e**) adsorption of a single F on Fe. Color code: grey is carbon, blue is nitrogen, orange is iron, and green is fluorine.

We first considered the adsorption of F_2_ on the Fe atom of the FeN_x_ sites. We found that F_2_ adsorbs on Fe for all the FeN_x_ configurations of Figure 1. When adsorbed on Fe, the F_2_ molecule is strongly stretched relative to the free F_2_ molecule. In the example of Figure 3a, where we use the basic structure of Figure 1j, the spacing between the two F atoms of the adsorbed F_2_ is 2.32 Å while the spacing of the two F atoms in the free F_2_ molecule is 1.11 Å. In fact, the adsorbed F_2_ molecule is subject to dissociation because, for example, the binding energy of the dissociated F_2_ molecule with one F atom adsorbed on Fe and the other F atom adsorbed on a near C atom either on the same side (Figure 3b) or on the opposite side (Figure 3c) of the carbon layer is, respectively, 1.36 eV and 1.40 eV lower. The fluorinated structure is considerably more stable when two F atoms are adsorbed on the Fe atom on opposite sides of the carbon layer (Figure 3d) because the energy of the system is 6.12 eV lower than that of F_2_ adsorbed on Fe (Figure 1a). The binding energies of the fluorine adsorbates shown in Figure 3 are given in Table 1. All F–Fe and F–C bond lengths appearing in Figure 3 are shown in Table A1 of the Appendix A.

**Figure 4:**
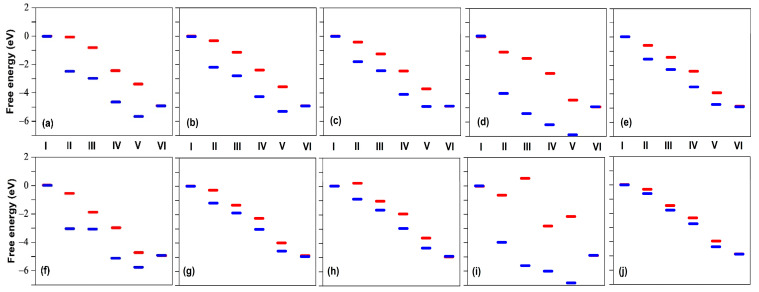
Relative free energy at zero potential for the six steps (I–VI) of the ORR sequence (1) for the atomic structures shown in Figure 1 with (red segments) and without (blue segments) a F atom adsorbed on Fe. The panels (**a**–**j**) correspond to the structures shown in Figure 1a to j.

In the case where two F atoms are adsorbed on Fe on both sides of the carbon layer (Figure 3d), the Fe site becomes unavailable to catalyze ORR. Indeed, the binding energy of the O_2_ molecule on the Fe atom of the pristine structure of Figure 3 is only −0.71 eV, meaning that the fluorinated iron sites of Figure 3d,e are very stable and the adsorbed F is unlikely to be spontaneously replaced by O_2_. The same conclusion holds for all structures of Figure 1, except for Figure 1d, for which the binding energy of Fe–O_2_ is stronger than that of Fe–F, as shown in Table 2. However, this structure appears to be inactive in the absence of fluorine, as can be seen in Figure 4d.

For a fluorinated FeN_x_ site located in a single carbon layer, the adsorption of a single F atom on the Fe site can turn a poor catalyst into an effective one due to the weakening of the binding energy of the adsorbates of the reaction sequence (1). This can be seen in Figure 4, which shows the relative free energies (with respect to the initial state) of each step of the catalytic reaction sequence (1) in the cases where the Fe sites are free of fluorine (blue segments) and where a F atom is adsorbed on Fe (red segments). One can see for the structures corresponding to Figure 4a,b,d,f, that the ORR catalytic process, which seems unlikely without the adsorption of F because of the very low energy level of step V, $*\mathrm{OH}+H_{2}O+\left(H^{+}+e^{-}\right)$, becomes thermodynamically viable with the adsorption of F on Fe. However, the structure corresponding to Figure 4i remains a poor catalyst with and without the adsorption of F on Fe. On the other hand, the structure corresponding to Figure 4h, which seems to be a possible good catalyst without fluorine, becomes less effective with fluorine because O_2_ can no longer adsorb on Fe (step II). Table A2 shows the F–Fe, O–Fe–O, and O–O bond lengths in O_2_, Fe–O_2_, and F–Fe–O_2_. It can be seen that the F–Fe bond length in F–Fe–O_2_ is almost the same as in Figure 3e (Table A1). However, the Fe–O and O–O bond lengths in F–Fe–O_2_ are slightly smaller than in Fe–O_2_, indicating that the Fe–O_2_ bond is weakened due to the presence of F, in agreement with the discussion above.

From this, we conclude that carbon-based catalysts with sufficient separation between carbon layers (which is equivalent to considering the catalytic sites located on a single carbon layer) could theoretically benefit from partial fluorination by the weakening of the adsorbate free energy. This effect leads to a more favorable free energy distribution for most of the fluorinated ORR active sites as compared to that of the sites without any F–Fe bond. The conclusion that ORR is generally promoted when an adsorbate is bound to the metal atom on the opposite side of the carbon plane was also obtained via the DFT calculations reported in [53,54].

### 2.2. Fluorination of the FeN_x_ Sites—Double Carbon Layer

The useful FeN_x_ active sites (those able to produce ORR) are actually thought to be mostly embedded at the surface of continuous graphene layer stacks or located between two discontinuous graphene layers of micro- or mesopores [20]. Therefore, the question naturally arises as to whether the ORR can take place between the first two carbon layers when a F atom is adsorbed on Fe on the more accessible free side of the FeN_x_ site. To answer this question, we considered a model using two carbon layers: on the top, a carbon layer containing the FeN_x_ moiety and, under this first layer, another one composed of a single graphene layer parallel to the first one and located at 3.6 Å from the first layer. We selected this interplanar distance, which is a little larger than that of d = 3.35 Å in graphite, because carbon is disorganized after the pyrolysis stage. As a matter of fact, a fairly broad distribution of d-spacings (between 3.5 and 4.1 Å) was measured for furnace turbostratic carbon black grades, regardless of their particle size and structure. The average TEM measured d-spacings range between 3.83 and 3.92 Å and are significantly larger than the X-ray measured d-spacings ranging from 3.52 to 3.56 Å [57]. Thus, we used the intermediate value of 3.6 Å.

**Figure 5:**
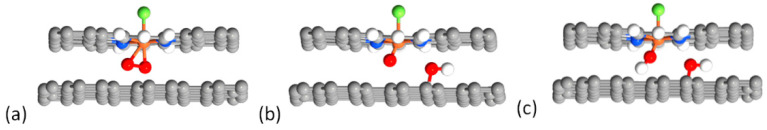
The first catalytic reaction steps between two carbon layers with a F atom adsorbed on Fe on the free side for the basic structure of Figure 1j. (**a**) Adsorption of O_2_ on Fe; (**b**) result of the spontaneous dissociation of OOH into O on Fe and OH on the opposite carbon layer; and (**c**) formation of OH adsorbed on Fe between the two layers. Color code: grey is carbon, blue is nitrogen, orange is iron, green is fluorine, and red is oxygen.

The first step in ORR is the adsorption of O_2_ on Fe. Then, according to the reaction sequence (1), each adsorbate combines with an H^+^ ion and an electron. Therefore, O_2_ and H^+^ have to migrate between the carbon layers to reach the Fe atom of the FeN_x_ site. This migration is certainly easier for porous carbon structures such as the one of Figure 1j or when the Fe atom is close to the edge of the carbon layer, as in Figure 1g,i. Figure 5 shows the basic structure of Figure 3e to which a parallel graphene plane was added under the graphene plane containing the F–FeN_x_ site. One notes that there is apparently enough room between the two carbon layers to accommodate an oxygen atom or molecule because the spacing between the carbon layers of our amorphous carbon catalyst is assumed here to be 3.6 Å, while the theoretical radii of Fe, C, and O are 1.56 Å, 0.67 Å, and 0.48 Å [58], respectively, so that the sum of the radius of Fe, the diameter of O_2_, and the radius of C is 3.19 Å, which is smaller than the assumed spacing between the carbon layers.

**Figure 6:**
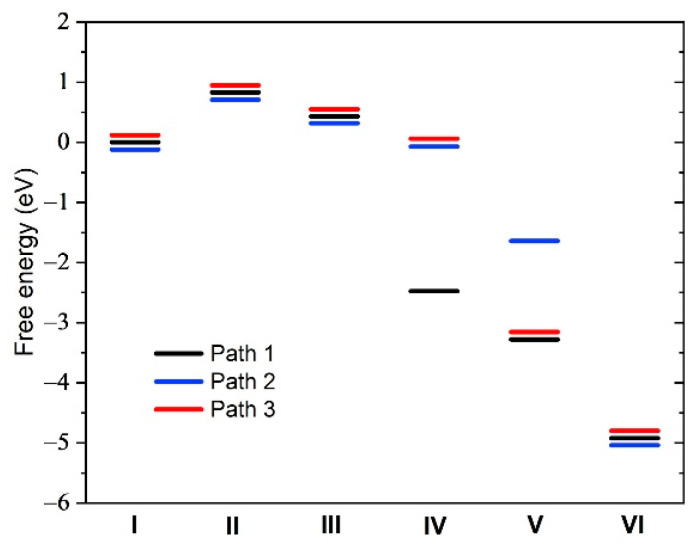
Relative free energy at zero potential for the six steps of the ORR sequences between two carbon layers for the structure of Figure 3e to which a parallel graphene plane was added under the graphene plane containing the F–FeN_x_ site. The three possible paths are explained in the text. The energy levels have been shifted slightly to facilitate path identification.

The free energy diagram of the catalytic reaction is shown in Figure 6. O_2_ can adsorb on the Fe atom between the planes (Figure 5a and step II in Figure 6) but it needs around 1 eV to get there. This is a consequence of the stress induced on the surrounding structure by the insertion of the O_2_ molecule. Our transition state calculation indicates that the activation energy is around 1.5 eV between the state of O_2_ in the pore and its adsorbed state on the Fe atom. However, from the latter adsorbed state (step II in Figure 6), a continuously decreasing free energy sequence can be found, but with modifications with respect to the sequence (1). When adding $H^{+}+e^{-}$ to the adsorbed O_2_, OOH spontaneously dissociates into an O adsorbed on Fe and an OH which adsorbs on a carbon atom of the opposite layer (Figure 5b and step III in Figure 6). The reactions up to step III in Figure 6 are as follows:

(2) $$\begin{array}{c} {\mathrm{Fe}+G+O}_{2}+4\left(H^{+}+e^{-}\right)\\ I \end{array}\begin{array}{c} \;\;\;\;\to \;\;{(\mathrm{Fe}-O}_{2})+G+\;4\left(H^{+}+e^{-}\right)\;\;\to \\ \;\;\;\;\;\;\;\mathrm{II} \end{array}\;\begin{array}{c} (\mathrm{Fe}-O)+(G-\mathrm{OH})+\;3\left(H^{+}+e^{-}\right)\;\;\\ \mathrm{III} \end{array}$$

where G is the bottom graphene plane. Then three distinct paths are possible, depending on how the successive $H^{+}+e^{-}$ are added to the adsorbates. These paths are illustrated in Figure 6 by black, blue, and red segments, respectively. The last step (VI) is ${\mathrm{Fe}+G+2H}_{2}O$, i.e., the formation of two water molecules after the exchange of four electrons. The steps IV and V for the three paths are, respectively,

(3) $$\;\;\begin{array}{c} \mathrm{Path}\;1:\;{(\mathrm{Fe}-O)+G+H}_{2}O+2\left(H^{+}+e^{-}\right)\;\;\to \\ \;\;\;\;\;\;\;\mathrm{IV} \end{array}\;\;\begin{array}{c} (\mathrm{Fe}-\mathrm{OH})+G+\;H_{2}O+\left(H^{+}+e^{-}\right)\\ V \end{array}$$

(4) $$\begin{array}{c} \mathrm{Path}\;2:\;(\mathrm{Fe}-\mathrm{OH})+(G-\mathrm{OH})+2\left(H^{+}+e^{-}\right)\;\to \;\\ \;\;\;\;\;\;\;\mathrm{IV} \end{array}\begin{array}{c} \mathrm{Fe}+(G-\mathrm{OH})+\;H_{2}O+\left(H^{+}+e^{-}\right)\;\;\\ V \end{array}$$

(5) $$\begin{array}{c} \mathrm{Path}\;3:\;(\mathrm{Fe}-\mathrm{OH})+(G-\mathrm{OH})+2\left(H^{+}+e^{-}\right)\to \\ \;\;\;\;\;\;\;\mathrm{IV} \end{array}\begin{array}{c} \;(\mathrm{Fe}-\mathrm{OH})+G+\;H_{2}O+\left(H^{+}+e^{-}\right)\;\;\\ V \end{array}$$

The most complex intermediate state, where one OH is adsorbed on Fe and the other OH is adsorbed on the opposite graphene layer G, is shown in Figure 5c and corresponds to step IV of paths 2 and 3.

**Figure 7:**
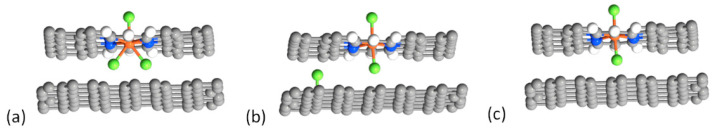
Adsorption of F_2_ and F on the Fe site between two carbon layers for the basic structure of Figure 1j. (**a**) Adsorption F_2_ on Fe; (**b**) adsorption of F on Fe and F on a nearby carbon site (dissociated form of F_2_); and (**c**) adsorption of F on Fe between two carbon layers.

Since the theoretical radius of F (0.42 Å) is smaller than that of O (0.48 Å) [58], F and F_2_ can also be accommodated between the two layers. We also performed DFT calculations for that case. The results are illustrated in Figure 7. The binding energies of F_2_ and F in the cases of Figure 7a,b are −1.84 and −4.48 eV, respectively. Here the reference structure is composed of the two planes with the external adsorbed F atom on Fe. Thus, as in the monolayer case of Figure 3, the dissociation of F_2_ is favored between the two layers of carbon. In addition, contrary to O_2_, the adsorption of F_2_ (or its dissociated form) between the plane is exothermic. For the sake of comparison with the single plane case of Figure 3d, the binding energy of the two F on both sides of the plane is −6.24 eV (Figure 7c) vs. −7.88 eV in the case of Figure 3d. Again, the difference in binding energy is due to the stress induced on the surrounding structure by the insertion of the F atom. The existence of F–Fe–F bonds in the catalyst could correspond to the specific peak at ~685.4 eV assigned to the adsorption of two F atoms on Fe in the F1s XPS spectrum of the catalyst [33].

From this study of the fluorinated bilayer configuration, it appears that ORR catalysis between two carbon layers is inefficient, primarily because O_2_ requires about 1 eV to occupy the catalytic site, although the subsequent reaction steps are thermodynamically viable. Furthermore, upon fluorination of the catalyst, F_2_ and F can occupy the catalytic site at a lower energy cost, thereby poisoning the FeN_x_ sites.

### 2.3. Fluorination of Metal-Free Sites

**Figure 8:**
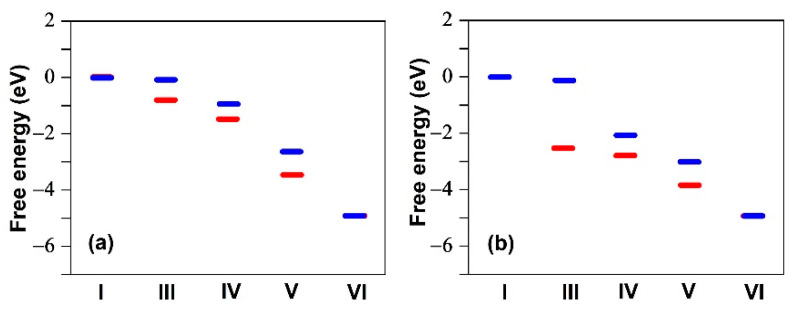
Relative free energy at zero potential for the six steps (I–VI) of the ORR sequence (1) for the sites shown in Figure 2 with (red segments) and without (blue segments) a F atom adsorbed on a carbon atom. (**a**) Armchair configuration of N-doped carbon; (**b**) zigzag configuration of N-doped carbon. The blue segments in (a) were obtained by considering the ORR activity of site 5 in Figure 2a. The blue segments in (b) were obtained by considering the ORR activity of site 1 in Figure 2b. The red segments in (a) were obtained by considering a F atom adsorbed on site 1 in Figure 2a, while keeping site 5 as ORR active. The red segments in (b) were obtained by considering a F atom adsorbed on site 1 in Figure 2b, while considering the ORR activity of site 6.

We now turn to the metal-free catalysts shown in Figure 2, which are also known to contribute to the ORR, but to a much lesser extent than the FeN_x_ metal sites free of fluorine [59]. It has been demonstrated that these catalysts can produce uniformly descending free energy steps for the reaction sequence (1), as for some of the FeN_x_ sites (see Figure 4). However, O_2_ hardly adsorbs on these structures [45,46,47,48,49]. Our DFT calculations are in good agreement with those previous works, as shown by the blue segments in Figure 8a,b, which were obtained by considering the ORR active sites 5 and 1 in Figure 2a,b, respectively.

We then verified whether the carbon catalytic sites can be poisoned by F or F_2_. For the armchair configuration of Figure 2a, adsorption of F on the sites numbered from 1 to 5 were tested. Table 3 shows that F can adsorb on the five sites with different binding energies. Site 1 has the strongest binding energy of −2.57 eV. When a F atom is adsorbed on site 1, Figure 8a (red segments) shows that the catalytic site 5 remains active.

For the zigzag structure we found that the catalytic site 1 in Figure 2b has the strongest binding energy of −3.78 eV for F. In this case, we carried out a calculation of the catalytic sequence on site 6 in Figure 2b. Even if the active site 1 is occupied by a fluorine atom, the ORR can take place on site 6, as can be seen in Figure 8b (red segments). Additional F–C bond length data for the five sites are given in Table A3. We note the anti-correlation between F–C bond length and binding energy as well as the smaller values compared to the F–Fe bond lengths given in Table A1.

We were unable to find a site where F_2_ could be adsorbed on both the armchair and zigzag structures, so that dissociation of F_2_ is unlikely on these structures. Therefore, F_2_ has to be dissociated elsewhere, such as on the Fe sites, for instance, or at any oxygenated functionality like COH, COOH, or C–O–C, known (by XPS) to be present at the surface of our (and many other) non-PGM catalysts [33]. These calculations tend to confirm that fluorination does not affect much the catalytic activity of the metal-free catalysts considered here, thus providing an explanation for the residual ORR catalytic activity found after fluorination up to a value of F/C = 0.27 (measured by NMR) of the fluorinated Fe/N/C catalyst observed in the experiments reported in [33].

### 2.4. Defluorination of the FeN_x_ Sites

Here we consider a question that has puzzled us for some time: the possibility to thermally de-fluorinate at 900 °C previously fluorinated FeN_x_ catalytic sites such as the ones illustrated, for instance, in Figure 3d,e. Let us label these configurations F–FeN_4_–F and F–FeN_4_, respectively. From Table 1, the binding energies of the fluorine adsorbates on the Fe atom are −4.56 eV for F–FeN_4_ and −7.88 eV for F–FeN_4_–F. Despite the high binding energies of these bonds, it was experimentally found that the latter are broken after a 30 min heat treatment at 900 °C in Ar of the fluorinated catalysts (no F1s XPS signal anymore, as seen in Figure 6A of [33]). How is this possible when the thermal energy at 900 °C is only around 0.1 eV?

It is certainly not because of the special nature of the catalytic sites such as those illustrated in Figure 3e,d, as Table 2 confirms that the FeN_x_ sites illustrated in Figure 1 are all characterized by Fe–F and F–Fe–F binding energies of several eV. De-fluorination is only possible if the fluorinated FeN_x_ sites are involved in reactions also involving either radicals or small molecules released from the catalyst surface under heat treatment. Reactions (6–9) below are examples of possible de-fluorination reactions. Our thermodynamic calculations show that all these reactions are characterized by a negative free energy change ΔG at 900 °C, meaning that they are spontaneous at that temperature.

(6) $${{(F-\mathrm{FeN}}_{4}–F)}_{\mathrm{solid}}+{\left(\mathrm{CH}•\right)}_{\mathrm{gas}}\;↔{\;(F-\mathrm{FeN}}_{4}{)}_{\mathrm{solid}}+C_{\mathrm{solid}\;\left(\mathrm{graphene}\right)}+{\left(\mathrm{HF}\right)}_{\mathrm{gas}}$$

(7) $${{(F-\mathrm{FeN}}_{4})}_{\mathrm{solid}}+{\left(\mathrm{CH}•\right)}_{\mathrm{gas}}\;↔{\;(\mathrm{FeN}}_{4}{)}_{\mathrm{solid}}+C_{\mathrm{solid}\;\left(\mathrm{graphene}\right)}+{\left(\mathrm{HF}\right)}_{\mathrm{gas}}$$

(8) $${{(F-\mathrm{FeN}}_{4}–F)}_{\mathrm{solid}}+{\left(\mathrm{CF}•\right)}_{\mathrm{gas}}\;↔{\;(F-\mathrm{FeN}}_{4}{)}_{\mathrm{solid}}+{{(\mathrm{CF}}_{2}•)}_{\mathrm{gas}}$$

(9) $${2(F-\mathrm{FeN}}_{4}–{F)}_{\mathrm{solid}}+{{(C}_{2}F_{6\;})}_{\mathrm{gas}}\;↔{\;2(F-\mathrm{FeN}}_{4}{)}_{\mathrm{solid}}+2{\left({\mathrm{CF}}_{4\;}\right)}_{\mathrm{gas}}$$

In these examples, ${\left(\mathrm{CH}•\right)}_{\mathrm{gas}}$, ${\left(\mathrm{CF}•\right)}_{\mathrm{gas}}$, and ${{(C}_{2}F_{6\;})}_{\mathrm{gas}}$ are decomposition products [60] generated at 900 °C from the carbonaceous or from the fluorinated carbonaceous supports of the catalysts. The evidence for the release of such gases is documented by the TGA curves already reported in several figures of [33] for these fluorinated catalysts.

## 3. Computational Methods

All DFT calculations reported here were done using the Vienna ab initio software package (VASP) [61,62,63,64]. The calculations were performed using the generalized gradient approximation (GGA) with the Perdew–Burke–Ernzerhof (PBE) functional [65]. The convergence criterion on the relative energy was set to 10^−5^ and the plane wave energy cut-off was set to 500 eV for all calculations. The Brillouin zone was sampled on regular 4 × 4 × 4 gamma grids. A graphene sheet with cell dimensions of a = 20.22 Å and b = 14.88 Å was used as a model for the carbon support. A void of 15 Å was included in the normal direction to avoid interactions between the periodic FeN_x_-doped carbon layers. The doped carbon structures were created by substituting carbon atoms of the graphene sheet by FeN_x_ groups or by N atoms in the case of metal-free catalysts. The positions of all atoms were fully relaxed, except in the case of the two carbon layers, where the positions of the carbon atoms were fixed to prevent the planes from moving relative to each other. However, for the calculation of the activation energy of O_2_ transiting between the two planes, in relation to Figure 5a, we used constraints where the edges of the planes were fixed along the *x*-axis while keeping the edges along the *y*-axis fixed, and vice versa. The activation energy was almost the same (1.5 eV) in both cases. The binding energy of an adsorbate on a given site was calculated using

(10) $$E_{catalyst+adsorbate}-\left(E_{\mathrm{catalyst}}+E_{adsorbate}\right)$$

where $E_{catalyst+adsorbate}$ is the energy of the carbon-doped catalyst with the adsorbate, $E_{catalyst}$ is the energy of the catalyst alone, and $E_{adsorbate}$ is the energy of the adsorbate far from the catalyst. The energy of $H^{+}+e^{-}$ is taken as half the energy of the H_2_ molecule, since H_2_ is at an equilibrium with its dissociated form $2\left(H^{+}+e^{-}\right)$ at the anode [43]. The molecules of the gas phases considered in this work, namely O_2_, H_2_, and F_2_, are assumed to be non-interacting with each other, which implies that only single molecules have been considered. Each step of the catalytic sequence (1) corresponds to a free energy given by

(11) $$G=G_{0}+ZPE+TdS+G_{\mathrm{sol}}$$

where $G_{0}$ is the energy of the structure per cell, *ZPE* is the zero point energy, *TdS* is the entropy term, and $G_{\mathrm{sol}}$ is the solvation energy arising by the aqueous medium. As was done in some of our previous works [52,54], for simplicity we assumed that the sum of the last three contributions nearly cancels, in agreement with [43,66]. However, corrections were brought to the intermediate state $*O+H_{2}O+2\left(H^{+}+e^{-}\right)$ and $*+2H_{2}O$ of the reaction sequence (1), which were inferred to be +0.4 and −0.6 eV, respectively [43].

## 4. Conclusions

We used DFT to examine the consequences of fluorination of the FeN_x_-doped and N-doped carbon catalysts used for ORR at the cathode of H_2_/O_2_ fuel cells. The main objectives of these calculations were to rationalize some of the experimental observations and to verify our conceptual representation of the catalytic sites and processes. We have considered several moieties of catalytic sites of FeN_x_-doped carbon with x ranging from 1 to 4. Most of them seem to be suitable catalysts for ORR because the free energy of the supposed catalytic sequence decreases regularly at zero potential. When the FeN_x_ sites are located on a single graphene layer, it turns out that F_2_ binds to Fe at FeN_x_ sites, with a binding energy of approximately −2 eV, but is subject to dissociation, leaving a single F on Fe with a binding energy of approximately −4 eV, which is stronger than the typical binding energy of O_2_ on Fe. In these conditions, ORR cannot happen on the F-poisoned FeN_x_ side, but is still possible on the other side of the F–FeN_x_ site, even transforming some otherwise poor un-poisoned FeN_x_ catalytic configurations into better F–FeN_x_ active ones. In addition, two F atoms can also bind to Fe on both sides of the carbon layer with almost twice the binding energy of a single F. When this happens, the Fe site is completely poisoned on both sides and is no longer able to catalyze ORR.

The occurrence of single graphene layers in actual catalysts is probably quite exceptional. Those are certainly better represented by several stacks of disorganized graphene layers forming a network of connected micropores and mesopores. Therefore, we have also examined the double graphene layer case where there is a second parallel carbon layer at a distance of 3.6 Å from the upper carbon layer carrying the FeN_x_ sites. We found that O_2_ adsorption on Fe between the two carbon layers is stable and that OOH dissociates spontaneously into O adsorbed on Fe and OH adsorbed on the opposite carbon layer. Because O_2_ adsorption increases the free energy by about 1 eV (and needs an activation energy of around 1.5 eV) relative to free O_2_, the catalytic process is unlikely in this case, even though the free energy of subsequent steps decreases monotonically. On the other hand, we found that F_2_ can adsorb on Fe between the two carbon layers without energy expenditure, making this process more likely than for O_2_. These results suggest complete poisoning of the FeN_x_ sites through extensive fluorination of the catalyst, in agreement with the experimental observations.

We then focused on the residual catalytic activity after fluorination by considering Fe-free N-doped carbon armchair and zigzag structures for which previous DFT calculations suggested a viable catalytic process although O_2_ hardly adsorbs on these structures. For both structures, the active catalytic site is a carbon atom near a N atom. We found that these catalytic structures are not poisoned by F or F_2_, thus justifying a residual ORR catalytic activity similar to that of the Fe-free catalysts observed for fluorinated Fe/N/C catalysts.

Finally, we provided an explanation for the recovery of ORR upon heating to 900 °C after fluorination. This explanation is based on the presence of radicals or small molecules released from the catalyst surface upon heat treatment. Most of the calculations presented in this work are based on free energy levels that only indicate whether a catalytic process is thermodynamically viable or not. A more thorough study would include the determination of activation energies. These calculations are very computationally demanding and will be the subject of future work.

## Figures and Tables

**Table 1:** Binding energies of the fluorine adsorbates for the structures shown in Figure 3.

Structure	Binding Energy (eV)
a	−1.76
b	−3.12
c	−3.16
d	−7.88
e	−4.56

**Table 2:** Binding energies in eV for F adsorbed on Fe (Fe–F), for two F adsorbed on Fe on both sides of the carbon layer (F–Fe–F), and for O_2_ adsorbed on Fe (Fe–O_2_) for the structures shown in Figure 1.

Structure	Fe–F	F–Fe–F	Fe–O_2_
a	−4.27	−12.10	−0.84
b	−4.00	−11.92	−2.50
c	−3.85	−11.88	−2.06
d	−3.94	−13.04	−4.60
e	−4.05	−12.02	−1.82
f	−4.66	−12.96	−3.50
g	−5.89	−12.94	−4.55
h	−3.18	−11.88	−1.37
i	−2.93	−10.40	−1.04
j	−4.56	−7.88	−0.71

**Table 3:** Binding energies in eV for a F atom adsorbed on the 5 carbon sites of Figure 2a (armchair) and Figure 2b (zigzag).

Structure	Site 1	Site 2	Site 3	Site 4	Site 5
Armchair	−2.57	−2.12	−2.33	−1.69	−2.18
Zigzag	−3.78	−3.52	−2.80	−2.36	−2.97

## Data Availability

Additional data are available upon request from the corresponding author. The data set generated by the simulations performed in this work has not been made publicly available due to its large quantity and diversity.

## Sample Availability

No Samples are available.

## Appendix A

**Table A1:** Lengths in Å of the bonds involving the F atoms in Figure 3a–e.

Structure/Bond	F–Fe	F–C	F–Fe–F
**a**	–	–	1.74, 1.98
**b**	1.77	1.66	–
**c**	1.76	1.64	–
**d**	–	–	1.78, 2.05
**e**	1.79	–	–

**Table A2:** Bond lengths in Å for free O_2_ and for adsorbates on the structure of Figure 1j.

Structure/Bond	F–Fe	O–Fe–O	OBO
**O_2_**	–	–	1.23
**Fe–O_2_**	–	1.88, 1.88	1.35
**F–Fe–O_2_**	1.80	1.92, 1.92	1.32

**Table A3:** F–C bond lengths in Å for sites 1 to 5 shown in the structures of Figure 2.

Structure/Position	Site 1	Site 2	Site 3	Site 4	Site 5
**Armchair**	1.51	1.56	1.52	1.69	1.53
**Zigzag**	1.44	1.49	1.53	1.67	1.50
